# Materialism, Body Surveillance, Body Shame, and Body Dissatisfaction: Testing a Mediational Model

**DOI:** 10.3389/fpsyg.2018.02088

**Published:** 2018-10-30

**Authors:** Qingqing Sun

**Affiliations:** College of Ideological and Political Education, Henan University of Economic and Law, Zhengzhou, China

**Keywords:** materialism, self-objectification, body shame, body dissatisfaction, Chinese women

## Abstract

The present study aimed to examine the mechanisms underlying the links between materialism and body dissatisfaction. A sample of 583 Chinese undergraduate women completed a questionnaire measuring materialism, body surveillance, body shame, and body dissatisfaction. Correlational analysis showed that materialism, body surveillance, and body shame were significantly positively correlated with body dissatisfaction. The results of path analyses revealed that higher materialism predicted more body dissatisfaction, albeit indirectly, via higher body surveillance and body shame.

## Introduction

Materialism refers to a value that emphasizes the importance of material possessions (Richins and Dawson, 1992). Numerous studies have demonstrated that materialism has negative psychological consequences in western countries (Manolis and Roberts, 2012; Dittmar et al., 2014) and China (Jiang et al., 2012; Li et al., 2016). Recent studies show that materialism is positively linked to women’s body dissatisfaction (Gunadóttir and Gararsdóttir, 2014), reminders of materialism makes women more dissatisfied with their bodies after exposure to thin-ideal media models (Ashikali and Dittmar, 2011), yet little is known about why this relationship exists. Therefore, this study intends to explore the mediating processes that explain how materialism influences body image among Chinese female college students.

A consumer culture impact model is often used to explain the relationship between materialism and body image concerns (Dittmar, 2008; Ashikali and Dittmar, 2011; Gunadóttir and Gararsdóttir, 2014). According to the consumer culture impact model (Dittmar, 2008), consumer culture is characterized by two predominant ideals: the body-perfect ideal and the material good life ideal. The body perfect ideal refers to being ultra-thin for women and ultra-muscular for men, and the material good life ideal refers to emphasis on affluence and luxurious possessions and lifestyles. These two cultural ideals are often closely linked, because they are typically shown together in the media. For example, in advertisements, people usually have both attractive looks and lavish lifestyles (Jiang et al., 2012). Therefore, a materialistic value orientation emphasizes looking beautiful as well as having money and expensive property (Ashikali and Dittmar, 2011). It suggests that people who endorse materialistic values will internalize the body-perfect ideal, thus, are more likely to experience body dissatisfaction. Indeed, Gunadóttir and Gararsdóttir (2014) found that materialism is positively correlated with the internalization of the thin- ideal for women, and materialism directly predicted the thin-oriented body dissatisfaction for women. Yet, no work has explored the mediating processes underlying materialism and body dissatisfaction.

Objectification theory offers a framework for understanding the processes that connect materialism and body dissatisfaction. According to objectification theory (Fredrickson and Roberts, 1997), women living in a culture that sexualizes the female body who then internalize an observer’s perspective on their own bodies, thus treating themselves as an object to be looked at and evaluated on the basis of appearance. This perspective, termed “self-objectification,” often manifests in body surveillance, or the individual habitually monitoring her outward appearance (Fredrickson and Roberts, 1997). In fact, in a consumerist society, women’s bodies are often commercialized and objectified. For example, in advertisements, an ideal woman’s body is often used as the selling point to attract attention and arouse people’s desire to consume (Cheng, 2015). Moreover, a woman’s perfect body is often associated with positive life outcomes in media (Luo, 2012), implicitly suggesting that having a perfect body can bring success and happiness. Therefore, it’s plausible that highly materialistic women will attach great importance to their physical appearance and are more likely to take an objectifying perspective on themselves and report monitoring of their bodies. Indeed, experimental and correlational research has found that materialism contributes to the development of women’s self-objectification (Teng et al., 2016a,b). Given that body surveillance is positively correlated with body image disturbance (Fitzsimmons-Craft et al., 2012; Jackson et al., 2015), therefore, we propose that materialism may influence Chinese college women’s body dissatisfaction via body surveillance.

**FIGURE 1:**
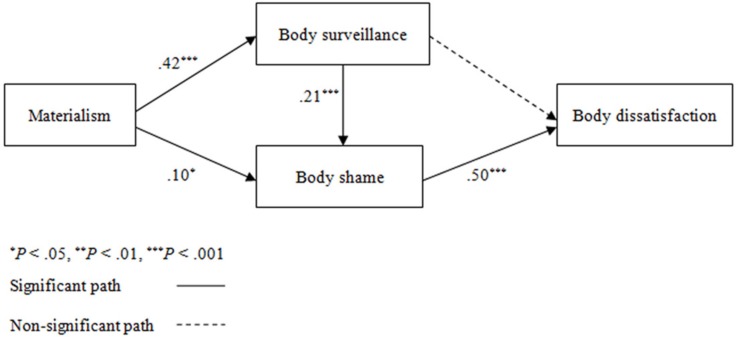
Hypothesized and final model with standardized path coefficients.

In addition, according to objectification theory, body surveillance can produce negative emotional experiences like body shame, the emotion that occurs when “people evaluate themselves relative to some internalized or cultural ideal and come up short” (Fredrickson and Roberts, 1997, p. 181). This in turn may contribute to various mental health risks such as body image disturbance and disordered eating (for a review, see Moradi and Huang, 2008). Therefore, we propose that materialism may contribute to body surveillance, and this in turn, may influence women’s body dissatisfaction via body shame (see Figure 1).

In sum, the three hypotheses tested in the current study were: materialism is positively correlated with body surveillance, body shame, and body dissatisfaction (Hypothesis 1); materialism predicts women’s body dissatisfaction through body surveillance (Hypothesis 2); materialism predicts women’s body dissatisfaction indirectly through higher self-objectification and then higher body shame (Hypothesis 3).

## Methods

### Participants and Procedures

Participants were 583 undergraduate women at a large university in Henan, China who completed the study for course credit. Participants had a mean age of 18.73 years (*SD* = 0. 76). The mean body mass index (BMI) of the sample was 20.10 kg/m^2^ (*SD* = 2.25). The Research Ethics Committee of Henan University of Economics and Law approved the study. All participants submitted online informed consent before filling in the questionnaire.

### Measurements

#### Demographic

Participants reported their age, ethnicity, height, and weight. Height and weight was used to calculate BMI.

#### Materialism

The 18-item Material Values Scale (Richins and Dawson, 1992) assessed participants’ endorsement of materialistic values. Participants responded on a seven-point Likert scale ranging from 1 (*totally disagree*) to 7 (*totally agree*). The scale has satisfactory reliability and validity in Chinese samples (e.g., Li and Guo, 2009). In this study, α = 0.78.

#### Body Surveillance

The eight-item Body Surveillance subscale of the Objectified Body Consciousness Scale (OBCS; McKinley and Hyde, 1996) assessed the frequency with which participants monitor their physical appearance. Items were scored on a seven-point Likert scale ranging from 1 (*strongly disagree*) to 7 (*strongly agree*). The scale has satisfactory reliability and validity in Chinese samples (e.g., Jackson et al., 2015). In this study, α = 0.76.

#### Body Shame

Body shame was assessed via six of eight Body shame subscale items (OBCS; McKinley and Hyde, 1996). The scale assessed the degree to which individuals feel shame about their bodies when they perceive themselves as not meeting the cultural standards of appearance. Items were rated from 1 (strongly disagree) to 7 (strongly agree). According to research on two Chinese undergraduate samples (e.g., Jackson et al., 2015), six of the eight Body shame subscale items were loaded on one component and significant correlations with body surveillance, eating disturbances and perceived appearance pressure from media were assessed. In the present study, α = 0.80.

#### Body Dissatisfaction

The 11-item Negative Physical Self-Scale-Fatness Scale (NPS-F, Chen et al., 2006) assessed thoughts, feelings, projections, and behaviors related to viewing the self as overweight. Items were scored on a five-point Likert scale ranging from 1 (Never like me) to 5 (Always like me). Sound reliability and validity have been reported in Chinese samples (e.g., Jackson et al., 2015). In the present study, α = 0.91.

## Results

Table 1 presents descriptive statistics for the study variables. As expected, indicators of materialistic values, body surveillance, body shame, and body dissatisfaction were all positively correlated.

**Table 1:** Descriptive statistics and correlation among study variables (*N* = 583).

Variables	*M*	*SD*	1	2	3	4
(1) Materialism	54.51	8.39	–			
(2) Body surveillance	33.81	7.29	0.48^∗∗^	–		
(3) Body shame	21.48	7.30	0.22^∗∗^	0.27^∗∗^	–	
(4) Body dissatisfaction	28.22	9.29	0.14^∗∗^	0.15^∗∗^	0.39^∗∗^	–


^∗^*P* < 0.05; ^∗∗^*P* < 0.01.

Path analysis was used to examine the relationships between materialism and body dissatisfaction. The proposed models were tested using AMOS 17.0 software (Figure 1). The results indicated an acceptable fit of the women’s data to the proposed model presented in Figure 1 (χ^2^= 7.577, *df* = 2, CFI = 0.994, TLI = 0.944, SRMR = 0.035, RMSEA = 0.069). However, the path from body surveillance to body dissatisfaction failed to attain significance (*p* < 0.05). An examination of modification indexes (MIs > 5.0) revealed that there was one additional significant path from materialism to body shame. Thus, we deleted the non-significant path and reanalyzed the model including the path from materialism to body shame and found that the fit statistics indicated a good fit to the data (χ^2^= 2.434, *df* = 2, CFI = 0.998, TLI = 0.996, SRMR = 0.0218, RMSEA = 0.019). At this point in the analysis, no path needed to be removed (see Figure 1). This revised model accounted for 15.5% of the variance in Chinese young women’s body dissatisfaction.

Next, we used the PROCESS (Model 6; Hayes, 2013) to further test the significance of each mediation effect proposed in Hypotheses 2 and 3. The significance of indirect paths was assessed using 95% bias corrected and accelerated confidence intervals with10000 bootstrap. Materialism was significantly related to higher body dissatisfaction indirectly through higher body shame (indirect effect = 0.05, *SE* = 0.02, 95% CI [0.01, 0.08]). Materialism was significantly related to higher body dissatisfaction indirectly through higher body surveillance and then higher body shame (indirect effect = 0.04, *SE* = 0.01, 95% CI [0.02, 0.06]). The direct effect ofmaterialism on body dissatisfaction was not significant after accounting for these mediators (direct effect = 0.08, *SE* = 0.05, 95% CI [−0.006, 0.17]).

## Discussion

In our modern consumerist society, a woman’s body is a capital that can manifest identity and social status and grant more social resources to change one’s life (Cheng, 2015). A perfect body means having more romantic relationships (Goldberg et al., 2003), more opportunities, and more success (Luo, 2012). Thus, highly materialistic women are in pursuit of a perfect body as well as wealth. For highly materialistic women, their bodies are more like commodities or objects to be evaluated on the basis of appearance, which is consistent with the concept of self-objectification. Therefore, this study based on the objectification theory investigated the association between materialism, body surveillance, body shame, and body dissatisfaction in a sample of young Chinese college women. We found that the endorsement of materialistic values predicts more body dissatisfaction, albeit indirectly, via higher body surveillance and body shame.

Contrary to our hypothesis, the mediating role of body surveillance between materialism and body dissatisfaction is not significant. This result was inconsistent with previous studies which found that body surveillance was a risk factor for body image disturbances (Lindner et al., 2012; Jackson et al., 2015). However, as expected, body surveillance mediated the association between materialism and body shame, and in turn body shame was associated with more body dissatisfaction. Given that materialistic values are strongly linked to the internalization of body-perfect ideals (Gunadóttir and Gararsdóttir, 2014), and that self-objectification is a cognitive process manifested by internalization of beauty ideals and body surveillance (Moradi, 2010), it is possible that highly materialistic woman constantly monitor their own bodies to compare them to a body-perfect ideal, leading them to feel shame (Knauss et al., 2008). Accordingly, our findings support and extend objectification theory (Fredrickson and Roberts, 1997), demonstrating that materialism influences women’s body dissatisfaction through a mechanism that have previously identified in the objectification literature.

Body shame mediated the association between materialism and body dissatisfaction. This result is was unexpected, but similar to previous findings (Knauss et al., 2008) which found that body shame mediated the internalization of ideals and body dissatisfaction. It suggests that women who endorse materialistic values have more body shame that may in turn contribute to body dissatisfaction. This result has important implications because body shame was a strong predictor of body image disturbances (Jackson et al., 2015) and disordered eating (Mehak et al., 2018). Therefore, future research is necessary to explore the full impact of materialism on woman’s body image.

Limitations of the current study should be considered. First, the present study was a cross-sectional design. Thus, no causal claims can be made about the relationships between the variables. Nonetheless, we believe that previous studies support the direction of our hypothesis. Experimental studies prove that materialism is an antecedent of self-objectification (Teng et al., 2016a), and self-objectification predicts greater body dissatisfaction (Jackson et al., 2015). Future research should implement studies with experimental and longitudinal designs to investigate potential causal relations between these variables. Second, the sample only consisted of Chinese college female students. Thus, the results may not be generalizable to other populations. However, research suggests that materialistic values predict men’s muscular-ideal body dissatisfaction (Gunadóttir and Gararsdóttir, 2014). Therefore, future research should examine this mediational mode among a larger and more diverse sample.

In conclusion, this study extends the body image literature by identifying a link between materialism and body dissatisfaction among young Chinese women. Our results showed that higher body surveillance and body shame are important mechanisms that help account for associations between materialism and body dissatisfaction.

## Author Contributions

QS carried out the experimental work and the data collection, interpretation, and wrote the manuscript.

## Conflict of Interest Statement

The author declares that the research was conducted in the absence of any commercial or financial relationships that could be construed as a potential conflict of interest. The reviewer AB and handling Editor declared their shared affiliation.
